# Knowledge, attitude, and practice toward oral frailty management among nursing staff and its determinant factors: a multicentre cross-sectional study

**DOI:** 10.3389/froh.2026.1824645

**Published:** 2026-07-22

**Authors:** Yanjie Zhang, Xijin Tan, Qiuxia Chen, Wenting Li, Zhixia Zhang, Siyi Chen, Tingting Zhou

**Affiliations:** 1 Department of Nursing, Tianyou Hospital Affiliated to Wuhan University of Science and Technology, Wuhan, Hubei, China; 2 School of Medicine, Wuhan University of Science and Technology, Wuhan, Hubei, China

**Keywords:** influencing factors, knowledge, attitude and practice, nursing education, nursing management, nursing staff, oral frailty

## Abstract

**Background:**

Oral frailty is an important component of frailty in older adults and is closely associated with multisystem functional decline. However, it has not been systematically integrated into routine nursing practice, and gaps remain in nursing staff's knowledge, attitude, and practice regarding oral frailty management. Examining nursing staff's knowledge, attitude, and practice and their influencing factors is essential for improving nursing competence and informing nursing education.

**Aims:**

To investigate the current status of knowledge, attitude, and practice regarding oral frailty management among nurses in general hospitals and to analyze the influencing factors, so as to provide evidence for the development of training programs to improve nurses' awareness and management ability related to oral frailty in China.

**Methods:**

A cross-sectional study was conducted using convenience sampling among 1,055 nursing staff from 15 general hospitals in Hubei Province, China. Data were collected using a self-developed questionnaire assessing nursing staff's knowledge, attitude, and practice regarding oral frailty management, which demonstrated acceptable reliability and validity. Univariate analyses and multiple linear regression were performed to examine levels of KAP and associated factors.

**Results:**

The mean scores for knowledge, attitude, and practice were 44.80 ± 12.77 (64.00%), 40.42 ± 8.67 (80.84%), and 34.47 ± 10.08 (68.94%), respectively. Multiple linear regression showed that being a geriatric specialist nurse, prior contact with patients with oral frailty, prior learning of relevant knowledge, and hospital level were associated with knowledge and attitude scores. Being a geriatric specialist nurse and having received relevant training were associated with practice scores. Years of working experience showed inconsistent effects across dimensions.

**Conclusion:**

The level of knowledge about oral frailty management among nurses was relatively low. Their attitudes were generally positive, but the implementation of related practices was insufficient. Training programs on oral frailty management knowledge and clinical practice should be developed according to the different characteristics of nurses. Such training may help promote the translation of knowledge into practice, improve nurses' ability in oral frailty management, and contribute to better oral health outcomes for older patients.

## 1 Introduction

Frailty is a multifaceted geriatric syndrome fundamentally linked to multi-system physiological decline, posing a significant public health challenge for the aging population (1). While traditional research has prioritized nutritional status and physical mobility, the emerging concept of “oral frailty” (OF) has gained traction as a critical determinant of healthy aging (2). OF refers to age-related decline in oral function accompanied by psychological and physical deterioration (3). It mainly includes reduced saliva secretion, tooth loss, swallowing dysfunction, poor oral hygiene, and chewing difficulties (4).

Studies have shown that older adults with oral frailty are more likely to develop physical frailty, sarcopenia, serious diseases requiring care, and even death compared with those without oral frailty (2). Beyond physical impairments, OF restricts social engagement and erodes mental well-being (5). For instance, its correlation with fall-related injuries can trigger a cycle of fear and social withdrawal, further diminishing independence (6). Previous research has confirmed that oral health-related factors play an important role in the development of frailty in older adults (7). Specifically, poor oral health practice, such as infrequent tooth brushing and low utilization of dental services, are significantly associated with increased risk of frailty (8). An increased number of missing teeth and greater need for dentures have also been found to raise the risk of frailty in later life (9). Self-reported oral pain and chewing difficulties are consistently associated with frailty status (10). In addition, clinical evidence shows that poor oral conditions and the need for monitoring oral changes are significantly related to higher frailty risk, suggesting that improving daily oral health practice may help prevent frailty (11).

In the management of oral frailty in older adults, nurses play an important role because they have frequent contact with patients and are responsible for health assessment and daily care. Although oral function decline has been recognized as part of frailty, it has not been fully included in routine clinical assessment, and multidisciplinary cooperation is still limited (12). At the same time, reviews have shown that oral health care is often neglected in hospitals and long-term care settings. Nurses may lack sufficient training and face difficulties in providing and monitoring oral care (13). Therefore, it is necessary to develop systematic training programs and improve nurses' ability in identifying and managing oral frailty, which may provide evidence for future educational interventions.

Based on the Knowledge–Attitude–Practice (KAP) theory, this study aims to investigate nurses' current situation in the identification, assessment, and management of oral frailty, and to analyze the related influencing factors, so as to provide evidence for developing targeted training and intervention programs.

## 2 Methods

### 2.1 Study design and participants

A cross-sectional survey was conducted using a convenience sampling method from August to October 2025 in 15 general hospitals in Hubei Province, China. A total of 1,055 nurses were included in the study. Since oral frailty predominantly affects older adults, nurses working in departments with a high proportion of older patients were selected to ensure the relevance of the study population.

The inclusion criteria were as follows: (1) at least 1 year of clinical work experience; (2) being a registered nurse working in geriatric-related departments, defined as departments in which ≥50% of hospitalized patients were aged 60 years or older according to hospital or nursing administration statistics; and (3) having the ability to understand and complete the questionnaire independently, voluntarily agreeing to participate, and providing informed consent. The exclusion criteria were: (1) nurses who were not on duty during the survey period due to personal leave, sick leave, or other reasons; and (2) nurse standardized training student, nursing interns, and other non-active staff.

The sample size was estimated according to Kendall's principle, which recommends a sample size of 5–10 times the number of questionnaire items for questionnaire-based studies. As the questionnaire contained 44 items (including 10 items on general characteristics and 34 items assessing knowledge, attitude, and practice regarding oral frailty management), and considering a potential 10%–20% rate of invalid or missing questionnaires, the required sample size was estimated to range from 242 to 528 participants. A total of 1,083 questionnaires were collected, of which 1,055 were valid, yielding an effective response rate of 97.4%. The final sample size substantially exceeded the minimum required sample size, thereby enhancing the statistical power and precision of the analyses.

### 2.2 Instruments

#### 2.2.1 General information questionnaire

A self-designed questionnaire was used to collect demographic and work-related information, including gender, age, years of working experience, highest educational level, professional title, department, whether the nurse was a geriatric specialist nurse, whether they had contact with patients with oral frailty, whether they had learned relevant knowledge about oral frailty, and hospital level.

#### 2.2.2 Oral frailty knowledge–attitude–practice questionnaire for nurses in general hospitals

The questionnaire used in this study was a previously developed and psychometrically validated instrument for assessing nurses' knowledge, attitudes, and practices regarding oral frailty management, based on the Knowledge–Attitude–Practice (KAP) model. The instrument was developed by the research team in a previous study and demonstrated satisfactory reliability and validity.

The questionnaire consisted of three dimensions: knowledge (14 items), attitude (10 items), and practice (10 items), with a total of 34 items. A 5-point Likert scale was used for all items. In the knowledge dimension, responses ranged from “very unfamiliar” to “very familiar”; in the attitude dimension, from “strongly disagree” to “strongly agree”; and in the practice dimension, from “never” to “always”. The knowledge dimension was designed to assess nurses' self-reported familiarity with and understanding of oral frailty-related concepts in clinical practice, rather than knowledge assessed through objective testing. Each item was scored from 1 to 5. The total score ranged from 34 to 170. Higher scores indicated higher levels of knowledge, more positive attitudes, and more frequent practice regarding oral frailty management. A standardized score was calculated using the formula: standardized score = (actual score/total possible score) × 100. The standardized score was used to describe the overall level of knowledge, attitudes, and practices regarding oral frailty management. No predefined cut-off values were applied to categorize participants into groups.

Reliability analysis showed that the overall Cronbach's *α* coefficient was 0.975. The Cronbach's *α* coefficients for the knowledge, attitude, and practice dimensions were 0.974, 0.982, and 0.981, respectively. The overall split-half reliability was 0.992, and the split-half reliability coefficients for the three dimensions were 0.982, 0.984, and 0.989, indicating good internal consistency and stability. Validity analysis showed that the scale-level content validity index (S-CVI) was 0.949, and the item-level content validity index (I-CVI) ranged from 0.830 to 1.000. Exploratory factor analysis demonstrated a KMO value of 0.957 and a significant Bartlett's test of sphericity (*χ*^2^ = 16,317.744, *P* < 0.001), indicating that the data were suitable for factor analysis and that the questionnaire structure was reasonable. Overall, the questionnaire showed good reliability and validity.

### 2.3 Data collection and quality control

Data were collected using an online questionnaire survey. The finalized questionnaire was uploaded to Wenjuanxing and Jianshu platforms to generate an electronic survey link. Before data collection, the principal investigator contacted the nursing departments of participating hospitals to explain the study purpose and obtain permission. With the support of nursing managers, the questionnaire link was distributed to nurses through departmental work groups. During the survey process, communication was maintained with each department to ensure that participants met the inclusion and exclusion criteria. Before completing the questionnaire, participants were provided with a unified explanation of the study purpose and instructions. Participation was entirely voluntary, and participants were informed that all responses would be treated confidentially and used solely for research purposes. Each IP address was allowed to submit the questionnaire only once, and all items were set as mandatory to ensure data completeness. After data collection, incomplete questionnaires were automatically excluded by the system. Subsequently, two trained graduate students independently reviewed the data. Any discrepancies in questionnaire screening were discussed and resolved through consensus before finalizing the dataset. Questionnaires with obvious errors, patterned responses, completion time less than 2 min, or logical inconsistencies were removed. Qualified questionnaires were entered into the database after verification to ensure data accuracy and quality.

### 2.4 Statistical analysis

Data entry and statistical analysis were performed using Excel 2019 and SPSS version 27.0. Categorical variables were described using frequency (*n*) and percentage (%). For continuous variables, data with normal distribution were expressed as mean ± standard deviation (*x* ± *s*), while non-normally distributed data were expressed as median and interquartile range [*M* (P25, P75)]. Group comparisons were conducted using independent-samples *t* test, one-way analysis of variance (ANOVA), or non-parametric tests (Mann–Whitney *U* test or Kruskal–Wallis *H* test), as appropriate. Multiple linear regression analysis was used for multivariate analysis. A two-sided *P* < 0.05 was considered statistically significant.

### 2.5 Ethical aspects

This study was approved by the Ethics Committee of Wuhan University of Science and Technology (Approval No. 2025203). All participants voluntarily took part in the study after providing informed consent.

## 3 Results

### 3.1 General characteristics of the participants

The general characteristics of the 1,055 participants are shown in Table 1.

**Table 1:** General characteristics of nurses and univariate analysis of oral frailty management knowledge, attitude and behavior scores (*n* = 1,055).

Variables	*N* (%)	Knowledge			Attitude			Practice		
		Score (mean ± SD)	*t*/*F*	*P*	Score (mean ± SD)	*t*/*F*	*P*	Score (mean ± SD)	*t*/*F*	*P*
Sex			−0.695	0.487		−0.98	0.327		0.33	0.741
Male	55 (5.2)	43.64 ± 14.29			39.31 ± 8.75			34.91 ± 10.46		
Female	1,000 (94.8)	44.87 ± 12.68			40.49 ± 8.67			34.45 ± 10.06		
Age (years)			7.899	<0.001		0.186	0.906		7.836	<0.001
≤25	253 (24.0)	47.82 ± 11.72			40.25 ± 8.01			36.77 ± 9.18		
26–35	474 (44.9)	44.58 ± 12.86			40.65 ± 9.03			34.42 ± 10.39		
36–45	237 (22.5)	43.19 ± 12.91			40.25 ± 8.55			33.03 ± 10.00		
≥46	91 (8.6)	41.76 ± 13.32			40.22 ± 8.97			32.11 ± 9.89		
Years of working experience (years)			5.998	<0.001		0.1	0.982		5.596	<0.001
≤5	352 (33.4)	47.37 ± 12.09			40.22 ± 8.38			36.16 ± 9.53		
6–10	236 (22.4)	44.11 ± 12.60			40.44 ± 8.66			34.71 ± 9.86		
11–15	231 (21.9)	43.82 ± 12.96			40.66 ± 9.22			33.76 ± 10.77		
16–20	104 (9.9)	43.24 ± 12.60			40.38 ± 8.27			31.68 ± 9.84		
≥21	132 (12.5)	42.14 ± 13.65			40.56 ± 8.88			32.99 ± 10.13		
Educational level			0.441	0.643		2.016	0.134		0.242	0.785
College diploma	212 (20.1)	45.23 ± 12.84			39.41 ± 8.86			34.89 ± 9.60		
Bachelor's degree	817 (77.4)	44.63 ± 12.77			40.65 ± 8.58			34.38 ± 10.19		
Master's degree or above	26 (2.5)	46.58 ± 12.57			41.73 ± 9.68			34.00 ± 10.64		
Professional title			4.173	0.002		1.028	0.392		4.152	0.018
Nurse	293 (27.8)	46.93 ± 12.47			40.60 ± 8.29			36.32 ± 9.53		
Senior nurse	307 (29.1)	44.60 ± 12.46			39.71 ± 8.87			34.31 ± 9.70		
Supervisor nurse	398 (37.7)	44.01 ± 13.06			40.69 ± 8.80			33.38 ± 10.90		
Associate chief nurse	54 (5.1)	40.69 ± 12.08			41.30 ± 8.64			32.98 ± 6.73		
Chief nurse	3 (0.3)	37.00 ± 20.22			45.33 ± 8.08			42.33 ± 13.28		
Department			0.924	0.464		1.948	0.084		1.691	0.134
Geriatrics	184 (17.4)	45.17 ± 13.76			41.78 ± 9.10			34.21 ± 10.88		
Rehabilitation	173 (16.4)	44.90 ± 11.96			40.09 ± 8.60			35.44 ± 9.62		
Neurology	183 (17.4)	44.48 ± 13.02			40.25 ± 8.39			33.56 ± 10.54		
Respiratory medicine	167 (15.8)	43.46 ± 11.50			39.46 ± 8.35			33.11 ± 9.62		
Cardiovascular medicine	178 (16.9)	44.47 ± 13.04			39.68 ± 8.44			35.22 ± 9.07		
Endocrinology	170 (16.1)	46.31 ± 13.09			41.22 ± 9.00			35.31 ± 10.46		
Geriatric specialty nurse			−4.145	<0.001		−4.384	<0.001		−2.847	0.004
No	826 (67.5)	43.95 ± 12.90			39.81 ± 8.80			34.01 ± 10.15		
Yes	229 (32.5)	47.87 ± 11.81			42.63 ± 7.81			36.14 ± 9.66		
Contact with oral frailty patients			40.295	<0.001		18.056	<0.001		3.932	0.048
No	846 (80.0)	43.58 ± 12.64			39.87 ± 8.82			34.17 ± 9.98		
Yes	209 (20.0)	49.73 ± 12.11			42.69 ± 7.68			35.71 ± 10.40		
Learning experience related to oral frailty			100.998	<0.001		16.779	<0.001		54.756	<0.001
None	567 (53.7)	40.30 ± 12.56			39.12 ± 9.34			31.81 ± 10.33		
Learned but not comprehensive	452 (42.8)	49.34 ± 10.66			41.74 ± 7.54			37.10 ± 8.73		
Systematically learned	36 (3.4)	58.64 ± 10.22			44.42 ± 7.44			43.53 ± 7.78		
Hospital level			6.713	0.01		18.763	<0.001		0.201	0.654
Tertiary hospital	712 (67.5)	45.51 ± 12.57			41.26 ± 8.19			34.56 ± 10.46		
Non-tertiary hospital	343 (32.5)	43.34 ± 13.08			38.69 ± 9.38			34.28 ± 9.23		

### 3.2 Scores of nurses' knowledge, attitude, and practice regarding oral frailty management and univariate analysis

Among the 1,055 nurses, the mean scores for the knowledge, attitude, and practice dimensions regarding oral frailty management were 44.80 ± 12.77, 40.42 ± 8.67, and 34.47 ± 10.08, respectively (Table 1). The corresponding scoring rates were 64.00%, 80.84%, and 68.94%.

Univariate analysis showed that, in the knowledge dimension, significant differences were found according to age, years of working experience, professional title, whether the nurse was a geriatric specialist nurse, whether they had contact with patients with oral frailty, whether they had learned relevant knowledge about oral frailty, and hospital level (*P* < 0.05). In the attitude dimension, significant differences were observed according to whether the nurse was a geriatric specialist nurse, whether they had contact with patients with oral frailty, whether they had learned relevant knowledge about oral frailty, and hospital level (*P* < 0.05). In the practice dimension, age, years of working experience, professional title, whether the nurse was a geriatric specialist nurse, whether they had contact with patients with oral frailty, and whether they had learned relevant knowledge about oral frailty were significantly associated with the scores (*P* < 0.05).

### 3.3 Multiple linear regression analysis of factors influencing nurses' knowledge, attitude, and practice regarding oral frailty management

The scores of the three dimensions of the oral frailty Knowledge–Attitude–Practice questionnaire were entered as dependent variables. Variables that showed statistical significance in the univariate analysis were considered as candidate independent variables for the multiple linear regression models. Considering the conceptual overlap among age, professional title, and years of working experience, multicollinearity was assessed using variance inflation factor (VIF). Age and years of working experience showed moderate collinearity (VIF = 5.164 and 5.805, respectively). Age was therefore excluded from the model. Professional title was not included in the final models because it overlaps conceptually with years of working experience as an indicator of nurses’ career stage. Years of working experience was included as the representative indicator of clinical experience in the final model. The independent variables were coded as follows. Years of working experience was treated as dummy variables, with ≤5 years as the reference group. The categories 6–10 years, 11–15 years, 16–20 years, and ≥21 years were coded as (1, 0, 0, 0), (0, 1, 0, 0), (0, 0, 1, 0), and (0, 0, 0, 1), respectively. Geriatric specialist nurse (no = 0, yes = 1); contact with patients with oral frailty (no = 0, yes = 1). For learning experience regarding oral frailty, “no learning” was used as the reference group; “learned but not comprehensively” was coded as (1, 0), and “systematically learned” as (0, 1). Hospital level was coded as non-tertiary hospital = 0 and tertiary hospital = 1. The results showed that being a geriatric specialist nurse, having contact with patients with oral frailty, having learned relevant knowledge about oral frailty, and hospital level were significant influencing factors of the knowledge dimension (Table 2). The same variables were identified as influencing factors of the attitude dimension (Table 3). For the practice dimension, being a geriatric specialist nurse and having learned relevant knowledge about oral frailty were significant influencing factors (Table 4). The effect of years of working experience varied across the three dimensions.

**Table 2:** Results of the multiple linear regression analysis of factors influencing nurses’ knowledge of oral frailty management.

Variables	*B* (95% CI)	SE	*β*	*t*	*P*
Constant	40.31 (38.48, 42.13)	0.928		43.438	<0.001
Years of working experience (years)					
6–10 vs. ≤5	−2.55 (−4.51, −0.58)	1.001	−0.083	−2.545	0.011
11–15 vs. ≤5	−1.93 (−3.92, 0.06)	1.015	−0.063	−1.905	0.057
16–20 vs. ≤5	−2.44 (−5.05, 0.17)	1.33	−0.057	−1.837	0.066
≥21 vs. ≤5	−3.50 (−5.89, −1.11)	1.219	−0.091	−2.873	0.004
Geriatric specialty nurse (yes vs. no)	3.30 (1.54, 5.06)	0.896	0.107	3.684	<0.001
Contact with oral frailty patients(yes vs. no)	2.22 (0.33, 4.11)	0.964	0.069	2.299	0.022
Learning experience related to oral frailty					
Partially learned vs. none	7.72 (6.16, 9.28)	0.796	0.299	9.704	<0.001
Systematically learned vs. none	16.57 (12.55, 20.58)	2.048	0.236	8.088	<0.001
Hospital level (tertiary vs. non-tertiary)	1.69 (0.18, 3.20)	0.768	0.062	2.198	0.028

Model fit: *R*^2^ = 0.186; adjusted *R*^2^ = 0.179; *F* = 26.594; *P* < 0.001.

**Table 3:** Results of the multiple linear regression analysis of factors influencing nurses’ attitude of oral frailty management.

Variables	*B* (95% CI)	SE	*β*	*t*	*P*
Constant	37.02 (36.00, 38.04)	0.52		71.146	<0.001
Geriatric specialty nurse (yes vs. no)	2.36 (1.12, 3.60)	0.632	0.112	3.731	<0.001
Contact with oral frailty patients(yes vs. no)	1.39 (0.03, 2.75)	0.693	0.064	2.002	0.046
Learning experience related to oral frailty					
Partially learned vs. none	2.01 (0.91, 3.12)	0.561	0.115	3.591	<0.001
Systematically learned vs. none	4.79 (1.91, 7.67)	1.467	0.1	3.264	0.001
Hospital level (tertiary vs. non-tertiary)	2.36 (1.26, 3.45)	0.557	0.127	4.23	<0.001

Model fit: *R*^2^ = 0.065; adjusted *R*^2^ = 0.06; *F* = 14.522; *P* < 0.001.

**Table 4:** Results of the multiple linear regression analysis of factors influencing nurses’ practice of oral frailty management.

Variables	*B* (95% CI)	SE	*β*	*t*	*P*
Constant	32.78 (31.55, 34.01)	0.626		52.392	<0.001
Years of working experience (years)					
6–10 vs. ≤5	−0.99 (−2.61, 0.63)	0.825	−0.041	−1.203	0.229
11–15 vs. ≤5	−1.38 (−3.02, 0.26)	0.837	−0.057	−1.647	0.1
16–20 vs. ≤5	−3.24 (−5.40, −1.09)	1.097	−0.096	−2.957	0.003
≥21 vs. ≤5	−1.90 (−3.87, 0.07)	1.005	−0.063	−1.894	0.059
Geriatric specialty nurse (yes vs. no)	1.92 (0.47, 3.37)	0.739	0.079	2.602	0.009
Contact with oral frailty patients(yes vs. no)	−0.83 (−2.38, 0.72)	0.79	−0.033	−1.05	0.294
Learning experience related to oral frailty					
Partially learned vs. none	5.00 (3.71, 6.28)	0.657	0.245	7.606	<0.001
Systematically learned vs. none	11.18 (7.87, 14.49)	1.688	0.202	6.623	<0.001

Model fit: *R*^2^ = 0.108; adjusted *R*^2^ = 0.101; *F* = 15.854; *P* < 0.001.

## 4 Discussion

Among the three dimensions of oral frailty (OF) management, the attitude dimension showed the highest standardized score rate (80.84%), whereas the knowledge and practice dimensions showed comparatively lower score rates (64.00% and 68.94%, respectively). Overall, the results reflected a pattern of “acceptable attitudes but limited knowledge and insufficient implementation.” This finding is consistent with previous studies on nurses’ oral care knowledge (14, 15). Although most nurses recognized the importance of OF management, their knowledge of assessment, management, and intervention remained insufficient, and related practices were not consistently implemented, suggesting the importance of more systematic training. In addition, although nurses generally held positive attitudes toward OF management, their knowledge was often fragmented rather than systematic. This may be associated with a gap between attitudes and clinical practice, suggesting that positive attitudes alone may not be sufficient to support the consistent implementation of OF-related practices. Heavy workloads, time constraints, and competing clinical priorities may further hinder the implementation of OF-related assessment and intervention measures in routine care. Previous studies have indicated that high-quality nursing practice results from the interaction and coordinated development of knowledge, attitudes, and practices, with knowledge serving as an important foundation for practice implementation (16). Without systematic and continuous knowledge support, even positive attitudes may not lead to standardized and sustainable practices (17). The relatively low knowledge level and insufficient practice observed in this study are consistent with these findings. Therefore, OF management should be incorporated into routine nursing management through relevant training programs, quality control systems, and standardized clinical workflows. Practice-oriented training and clear assessment and intervention guidance may facilitate the translation of knowledge and attitudes into clinical practice, thereby promoting its sustainable implementation.

The results of this study showed that years of working experience had limited and inconsistent associations with knowledge and practice related to oral frailty (OF) management. In the multiple linear regression analysis, using ≤5 years of working experience as the reference group, nurses with 6–10 and ≥21 years of experience had significantly lower scores in the knowledge dimension. In the practice dimension, nurses with 16–20 years of experience also showed significantly lower scores. However, most categories of years of working experience were not statistically significant, and no clear dose–response pattern was observed. These findings suggest that an increase in years of working experience does not necessarily correspond to higher knowledge and practice levels related to OF management, which differs from some previous studies (18–20). From a clinical perspective, OF often has an insidious onset, non-specific manifestations, and may be easily confused with normal aging (21). This requires nurses to have strong abilities in risk identification and standardized management to identify risks, conduct assessments, and implement standardized management. Some studies have reported that junior nurses may have more recent exposure to new concepts, tools, and updated evidence during pre-service training and standardized education, which may give them a “recent learning advantage” in the knowledge dimension (22). In contrast, nurses with longer working experience may undertake greater clinical and managerial responsibilities, leaving less time for continuing education and potentially affecting the adoption of new knowledge and practices. Therefore, stratified training and assessment strategies should be developed according to nurses' experience levels. Targeted continuing education and practical support may enhance nurses' competence in OF identification, assessment, and intervention, thereby promoting standardized practice and improving management quality.

Nurses who had contact with patients with oral frailty (OF) scored higher in both the knowledge and attitude dimensions than those who had no such contact. This suggests that clinical exposure may be associated with better knowledge and attitudes regarding OF management. Previous KAP studies conducted in different clinical fields have also reported that clinical experience is positively associated with higher levels of knowledge and more positive attitudes (23). This finding may be explained by the idea that caregiving experience may provide opportunities for experiential learning. OF management involves multiple aspects, including chewing, swallowing, nutritional risk, oral hygiene, and functional training. Its manifestations are often subtle and may coexist with underlying diseases (24). During clinical practice, nurses may become more aware of gaps in their knowledge and skills and actively seek support from multidisciplinary team members and relevant resources, which may facilitate acquisition of OF-related knowledge. Therefore, experience in caring for patients with OF may be associated with greater awareness and understanding of OF management. These findings suggest that training programs should consider differences in clinical exposure and incorporate practice-oriented learning approaches to promote improvements in nurses' knowledge and attitudes.

Hospital level was significantly associated with nurses' knowledge and attitude toward oral frailty (OF) management. Nurses working in tertiary hospitals had significantly higher scores than those in non-tertiary hospitals. This suggests that differences in hospital level may reflect variations in training resources and clinical exposure in shaping nurses' understanding of OF. Previous studies have shown that nurses in tertiary hospitals generally demonstrate better specialty nursing knowledge and more positive attitudes than those in secondary hospitals (25). In addition, related research has indicated that nurses' knowledge and attitudes may be influenced by institutional resources and training systems (26). Hospital level may, to some extent, reflect differences in educational support, continuing education systems, and organizational structure. Tertiary hospitals generally undertake more teaching and research responsibilities, have more established continuing education systems, and place greater emphasis on multidisciplinary collaboration in complex case management. These differences may facilitate exposure to new concepts and standardized management approaches, which may be associated with higher knowledge and attitudes (27–29). At the same time, tertiary hospitals often manage more complex clinical conditions. In daily practice, nurses are more likely to encounter swallowing disorders, malnutrition, and decline in oral function, all of which are closely related to OF. Previous studies have suggested that clinical exposure may be associated with improved risk identification ability and professional knowledge, and may also increase their awareness of management needs, encouraging them to pay greater attention to relevant issues (30, 31). Therefore, differences in training resources and practice environments across hospital levels should be considered when promoting competence in OF management. Strengthening training and multidisciplinary support in non-tertiary hospitals may help reduce disparities in OF-related knowledge and attitudes among nurses.

Geriatric specialist nurse status and OF-related learning were key correlates of nurses' knowledge, attitudes, and practices regarding OF management (*P* < 0.05). Geriatric specialist nurses scored higher in all three dimensions compared with non-specialist nurses, suggesting that specialized geriatric nursing training may be associated with better understanding and practice ability in OF management. Previous studies have shown that systematic geriatric nursing education has been reported to improve nurses' knowledge of age-related health issues and their attitudes toward care, thereby promoting changes in clinical behavior (32, 33). In addition, nurses who had learned knowledge related to OF achieved higher scores, indicating that structured learning may strengthen recognition of the importance of OF management and facilitate the translation of knowledge into practice. This process is consistent with the theoretical pathway of the Knowledge–Attitude–Practice (KAP) model (34). As an emerging geriatric syndrome, OF has attracted increasing attention in recent years and is closely associated with physical frailty and other adverse outcomes (5, 35). Its management involves assessment, oral care, and multidimensional intervention strategies (36). Therefore, systematic training may be important for nurses' competence in OF management and promoting standardized practice. Based on these findings, OF-related content should be incorporated into continuing education and in-service training programs. Particular attention should be given to nurses without systematic training, and targeted educational support may help address gaps in OF-related assessment and intervention competencies. Furthermore, regular evaluation of training outcomes may help strengthen nurses' knowledge, attitudes, and practices, thereby supporting the continuity and quality of OF management.

## 5 Study strengths and limitations

This study has several limitations. First, due to the cross-sectional design, the findings primarily reflect associations between variables, and causal relationships cannot be established. Second, convenience sampling was adopted, and participants were recruited from hospitals within a single province, which may limit the representativeness and generalizability of the findings. In addition, the exact volume of older adults attending each participating hospital was not collected; therefore, potential differences in nurses' exposure to oral frailty management across hospitals could not be evaluated. Third, the data were collected through self-reported questionnaires, which may be subject to recall bias and social desirability bias. Although anonymous surveys were used to encourage honest responses, these biases cannot be completely excluded. Moreover, because the knowledge dimension was based on self-reported familiarity with and understanding of oral frailty-related concepts, findings related to knowledge should be interpreted as perceived knowledge rather than objectively tested knowledge. Future studies may incorporate objective knowledge assessments and further evaluate the questionnaire, including its test–retest reliability, in broader populations and settings. Longitudinal or interventional studies are also warranted to further examine the effects of oral frailty-related education or training on nurses' competence and related clinical outcomes.

## 6 Conclusion

This study indicated that nurses' knowledge and practice levels regarding oral frailty (OF) management remain relatively insufficient. Although overall attitudes were generally positive, practical management capacity still needs improvement. A geriatric specialist background, contact with patients with OF, prior learning of OF-related knowledge, and hospital level were important factors associated with nurses' knowledge and attitudes. A geriatric specialist background and relevant training experience were common factors associated with practice performance, while the effect of years of working experience varied across different dimensions. Systematic training appears to be an important factor associated with nurses' competence in OF management. The findings suggest that future OF-related educational initiatives may particularly benefit nurses without prior OF-related learning experiences, nurses working in non-tertiary hospitals, and those with limited exposure to patients with OF. Integrating OF-related assessment and management content into continuing nursing education and providing opportunities for practice-oriented learning may be associated with reduced gaps in knowledge and practice. In addition, strengthening institutional support and multidisciplinary collaboration may be important contextual factors for promoting standardized OF management in clinical settings.

## Data Availability

The raw data supporting the conclusions of this article will be made available by the authors, without undue reservation.
