# Supplementary material for: Knowledge, attitude, and practice toward oral frailty management among nursing staff and its determinant factors: a multicentre cross-sectional study
Source: Front Oral Health. 2026 Jul 22;7:1824645. doi: 10.3389/froh.2026.1824645 (PMC13437640; doi:10.3389/froh.2026.1824645)
Supplement: Supplementary file 1 [file Datasheet1.docx]

**Section A. Knowledge Dimension**

This section consists of 14 items covering key aspects of oral frailty management, including conceptual overview, risk factors, clinical manifestations, adverse health outcomes, identification and assessment methods, and prevention and management strategies.

Please select the option that best reflects your level of familiarity with oral frailty–related knowledge in the care of hospitalized older adults.

K1. Oral frailty (OF) refers to a condition in older adults characterized by age-related tooth loss, decline in oral function, and deterioration of oral hygiene, which may progressively lead to difficulties in eating and subsequent physical and psychological functional decline.

☐ Very unfamiliar ☐ Unfamiliar ☐ Moderately familiar ☐ Familiar ☐ Very familiar

K2. The early stage of oral frailty refers to a warning phase characterized by mild decline in oral function or the presence of potential risk factors and is considered potentially reversible, whereas the advanced stage represents severe deterioration of oral function.

☐ Very unfamiliar ☐ Unfamiliar ☐ Moderately familiar ☐ Familiar ☐ Very familiar

K3. In China, the prevalence of oral frailty among older adults is relatively high, particularly in rural areas. How familiar are you with this epidemiological situation?

☐ Very unfamiliar ☐ Unfamiliar ☐ Moderately familiar ☐ Familiar ☐ Very familiar

K4. The clinical manifestations of oral frailty include decreased chewing and swallowing ability, reduced salivary secretion, loose or missing teeth, tongue muscle weakness, unclear speech, slow eating, preference for soft foods, oral discomfort, reduced social interaction, and malnutrition.

☐ Very unfamiliar ☐ Unfamiliar ☐ Moderately familiar ☐ Familiar ☐ Very familiar

K5. Early symptoms of oral frailty, such as slightly slower eating, mild reductions in speech clarity, and mild oral dryness, are often subtle and non-painful. As a result, they may be easily overlooked by older adults and their caregivers and are frequently misattributed to normal aging, leading to missed opportunities for early intervention.

☐ Very unfamiliar ☐ Unfamiliar ☐ Moderately familiar ☐ Familiar ☐ Very familiar

K6. Oral frailty may affect systemic health through mechanisms such as inflammation and nutriregulation, and is associated with increased risks of physical frailty, pneumonia, reduced bone density, malnutrition, cardiovascular disease, and diabetes, as well as a higher risk of cognitive impairment and depression.

☐ Very unfamiliar ☐ Unfamiliar ☐ Moderately familiar ☐ Familiar ☐ Very familiar

K7. Oral frailty and systemic diseases (e.g., diabetes, cardiovascular disease, and cognitive impairment) are linked by a bidirectional vicious cycle, whereby systemic diseases can worsen oral function and oral frailty can negatively affect overall health.

☐ Very unfamiliar ☐ Unfamiliar ☐ Moderately familiar ☐ Familiar ☐ Very familiar

K8. Risk factors for oral frailty include physiological (e.g., advanced age, chronic illness, malnutrition), behavioral (e.g., smoking, polypharmacy, chemotherapy history), and psychosocial factors (e.g., low self-efficacy and limited social support).

☐ Very unfamiliar ☐ Unfamiliar ☐ Moderately familiar ☐ Familiar ☐ Very familiar

K9. Oral frailty is mainly assessed using functional indicators (e.g., chewing, swallowing, salivary secretion, remaining teeth, and tongue function), together with questionnaire assessments, functional tests, and imaging examinations.

☐ Very unfamiliar ☐ Unfamiliar ☐ Moderately familiar ☐ Familiar ☐ Very familiar

K10. Common tools for assessing oral frailty include OFI-8, OFI-6, OFST, and OHAT. An OFI-8 score of 1–3 suggests prefrailty, while a score of ≥4 suggests oral frailty.

☐ Very unfamiliar ☐ Unfamiliar ☐ Moderately familiar ☐ Familiar ☐ Very familiar

K11. A balanced diet is important for preventing oral frailty. Older adults should consume sufficient protein, vitamins, and minerals and avoid prolonged reliance on soft or high-sugar diets to maintain nutrition and oral function.

☐ Very unfamiliar ☐ Unfamiliar ☐ Moderately familiar ☐ Familiar ☐ Very familiar

K12. Good oral hygiene practices (e.g., proper brushing, use of dental floss or oral irrigators, and regular dental cleaning) can reduce the risk of periodontal disease and tooth loss, thereby lowering the risk of oral frailty.

☐ Very unfamiliar ☐ Unfamiliar ☐ Moderately familiar ☐ Familiar ☐ Very familiar

K13. Regular oral health check-ups, timely management of tooth loss and oral infections, and appropriate oral functional training can help slow the progression of oral frailty.

☐ Very unfamiliar ☐ Unfamiliar ☐ Moderately familiar ☐ Familiar ☐ Very familiar

K14. Comprehensive oral functional training (e.g., oral exercises, tongue resistance, speech, chewing, and swallowing training) can strengthen oral muscles, improve chewing and swallowing function, and reduce the risk of eating difficulties and aspiration.

☐ Very unfamiliar ☐ Unfamiliar ☐ Moderately familiar ☐ Familiar ☐ Very familiar

**Section B. Attitude Dimension**

This section consists of 10 items focusing on nurses’ attitudes, risk perception, and professional beliefs regarding oral frailty management.

Please select the option that best reflects your attitudes and beliefs toward oral frailty (OF) in the care of hospitalized older adults.

A1. I believe that mastering and continuously updating knowledge related to oral frailty is essential for improving the quality and professionalism of geriatric nursing care.

☐ Strongly disagree ☐ Disagree ☐ Neutral ☐ Agree ☐ Strongly agree

A2. I believe that hospitals or clinical departments should regularly provide systematic training on oral frailty.

☐ Strongly disagree ☐ Disagree ☐ Neutral ☐ Agree ☐ Strongly agree

A3. I believe that nurses should possess basic competencies in assessing and identifying oral frailty, as this is an essential part of our professional responsibility.

☐ Strongly disagree ☐ Disagree ☐ Neutral ☐ Agree ☐ Strongly agree

A4. I believe that integrating oral frailty assessment and management into routine nursing care is both feasible and necessary.

☐ Strongly disagree ☐ Disagree ☐ Neutral ☐ Agree ☐ Strongly agree

A5. I am concerned about eating or communication difficulties experienced by older adults due to oral problems, and I believe that these issues deserve a high level of attention.

☐ Strongly disagree ☐ Disagree ☐ Neutral ☐ Agree ☐ Strongly agree

A6. I believe that scientifically grounded nursing interventions can effectively prevent or improve oral frailty in older adults.

☐ Strongly disagree ☐ Disagree ☐ Neutral ☐ Agree ☐ Strongly agree

A7. I am willing to actively participate in multidisciplinary collaboration to jointly develop comprehensive care plans for managing oral frailty in older adults.

☐ Strongly disagree ☐ Disagree ☐ Neutral ☐ Agree ☐ Strongly agree

A8. I believe that actively monitoring and documenting changes in oral function (such as chewing and swallowing) and oral hygiene in older patients is important in daily nursing practice.

☐ Strongly disagree ☐ Disagree ☐ Neutral ☐ Agree ☐ Strongly agree

A9. Successfully helping patients improve oral health problems gives me a strong sense of professional accomplishment and professional value.

☐ Strongly disagree ☐ Disagree ☐ Neutral ☐ Agree ☐ Strongly agree

A10. I expect that hospitals will provide institutional and resource support to facilitate the implementation of oral frailty management in nursing practice.

☐ Strongly disagree ☐ Disagree ☐ Neutral ☐ Agree ☐ Strongly agree

**Section C. Practice Dimension**

This section consists of 10 items addressing nurses’ clinical practices related to the prevention and management of oral frailty in older adults, including knowledge acquisition, risk assessment, nursing interventions, multidisciplinary collaboration, and health education.

Please select the option that best reflects your actual behaviors in daily clinical practice.

P1. I use standardized assessment tools (such as the Oral Frailty Index-8 [OFI-8]) to assess the risk of oral frailty in older patients and regularly monitor changes over time.

☐ Never ☐ Rarely ☐ Sometimes ☐ Often ☐ Always

P2. For older patients at high risk of oral frailty (such as those who are bedridden or malnourished), I proactively implement preventive nursing measures.

☐ Never ☐ Rarely ☐ Sometimes ☐ Often ☐ Always

P3. I provide guidance to patients on oral functional training (such as chewing exercises and tongue muscle exercises) and regularly evaluate the effectiveness of these interventions.

☐ Never ☐ Rarely ☐ Sometimes ☐ Often ☐ Always

P4. I promptly communicate oral frailty–related oral conditions to physicians and participate in multidisciplinary teams to develop and implement individualized nursing care plans.

☐ Never ☐ Rarely ☐ Sometimes ☐ Often ☐ Always

P5. In my routine nursing care, I actively implement targeted measures to prevent the occurrence of oral frailty or to slow its progression in older patients.

☐ Never ☐ Rarely ☐ Sometimes ☐ Often ☐ Always

P6. I use educational materials such as brochures, demonstrations, or videos to guide patients and their family members in dietary management and oral hygiene maintenance.

☐ Never ☐ Rarely ☐ Sometimes ☐ Often ☐ Always

P7. I actively communicate and collaborate with colleagues such as physicians, dietitians, and rehabilitation therapists to jointly manage oral health problems in older patients.

☐ Never ☐ Rarely ☐ Sometimes ☐ Often ☐ Always

P8. I actively seek to learn about oral frailty–related knowledge and nursing strategies and apply them in clinical practice.

☐ Never ☐ Rarely ☐ Sometimes ☐ Often ☐ Always

P9. I actively share knowledge, new concepts, and clinical experience related to oral frailty with my colleagues.

☐ Never ☐ Rarely ☐ Sometimes ☐ Often ☐ Always

P10. I continuously enhance my professional competence in oral frailty management by participating in training programs or academic lectures.

☐ Never ☐ Rarely ☐ Sometimes ☐ Often ☐ Always

**You have now completed the questionnaire. Thank you sincerely for your time and cooperation.**
